# Neurocognitive and social-communicative function of children born very preterm at 10 years of age: Associations with microorganisms recovered from the placenta parenchyma

**DOI:** 10.1038/s41372-019-0505-8

**Published:** 2019-10-17

**Authors:** Martha Scott Tomlinson, Hudson P. Santos, Jill R. Stewart, Robert Joseph, Alan Leviton, Andrew B. Onderdonk, Karl C. K. Kuban, Timothy Heeren, T. Michael O’Shea, Rebecca C. Fry, for the ELGAN Study Investigators, Bhahvesh Shah, Rachana Singh, Linda Van Marter, Camilla Martin, Janice Ware, Cynthia Cole, Ellen Perrin, Frank Bednarek, Jean A. Frazier, Richard Ehrenkranz, Jennifer Benjamin, T. Michael O’Shea, Carl Bose, Diane Warner, Steve Engelke, Mariel Poortenga, Steve Pastyrnak, Padu Karna, Nigel Paneth, Madeleine Lenski, Michael Schreiber, Scott Hunter, Michael Msall, Danny Batton, Judith Klarr, Karen Christianson, Deborah Klein, Maureen Pimental, Collen Hallisey, Taryn Coster, Ellen Nylen, Emily Neger, Kathryn Mattern, Lauren Venuti, Beth Powers, Ann Foley, Joanne Williams, Elaine Romano, Debbie Hiatt, Nancy Peters, Patricia Brown, Emily Ansusinha, Gennie Bose, Janice Wereszczak, Janice Bernhardt, Joan Adams, Donna Wilson, Nancy Darden-Saad, Dinah Sutton, Julie Rathbun, Karen Miras, Deborah Weiland, Grace Yoon, Rugile Ramoskaite, Suzanne Wiggins, Krissy Washington, Ryan Martin, Barbara Prendergast, Beth Kring, Anne Smith, Susan McQuiston, Samantha Butler, Rachel Wilson, Kirsten McGhee, Patricia Lee, Aimee Asgarian, Anjali Sadhwani, Brandi Henson, Cecelia Keller, Jenifer Walkowiak, Susan Barron, Alice Miller, Brian Dessureau, Molly Wood, Jill Damon-Minow, Elaine Romano, Linda Mayes, Kathy Tsatsanis, Katarzyna Chawarska, Sophy Kim, Susan Dieterich, Karen Bearrs, Ellen Waldrep, Jackie Friedman, Gail Hounshell, Debbie Allred, Rebecca Helms, Lynn Whitley, Gary Stainback, Lisa Bostic, Amanda Jacobson, Joni McKeeman, Echo Meyer, Steve Pastyrnak, Joan Price, Megan Lloyd, Susan Plesha-Troyke, Megan Scott, Katherine M. Solomon, Kara Brooklier, Kelly Vogt

**Affiliations:** 1 Department of Environmental Sciences and Engineering, Gillings School of Global Public Health, University of North Carolina, Chapel Hill, NC USA; 2 School of Nursing, University of North Carolina, Chapel Hill, NC USA; 3 Department of Anatomy and Neurobiology, School of Medicine, Boston University, Boston, MA USA; 4 Neuroepidemiology Unit, Department of Neurology, Boston Children’s Hospital, Harvard Medical School, Boston, MA USA; 5 Department of Pathology, Harvard Medical School and Brigham and Women’s Hospital, Boston, MA USA; 6 Division of Pediatric Neurology, Department of Pediatrics, Boston Medical Center, Boston, MA USA; 7 Department of Biostatistics, Boston University School of Public Health, Boston, MA USA; 8 Department of Pediatrics, School of Medicine, University of North Carolina, Chapel Hill, NC USA; 9 Baystate Medical Center, Springfield, MA USA; 10 Boston Children’s Hospital, Boston, MA USA; 11 Tufts Medical Center, Boston, MA USA; 12 University of Massachusetts Medical School, Worcester, MA USA; 13 Yale University School of Medicine, New Haven, CT USA; 14 Wake Forest University, Winston-Salem, NC USA; 15 University of North Carolina, Chapel Hill, NC USA; 16 East Carolina University, Greenville, NC USA; 17 Helen DeVos Children’s Hospital, Grand Rapids, MI USA; 18 Sparrow Hospital, East Lansing, MI USA; 19 University of Chicago Medical Center, Chicago, IL USA; 20 William Beaumont Hospital, Royal Oak, MI USA; 21 Wake Forest University Baptist Medical Center, Winston-Salem, NC USA; 22 University Health Systems of Eastern Carolina, Greenville, NC USA

**Keywords:** Infection, Risk factors

## Abstract

**Objective:**

Infection of the placenta has been associated with preterm birth as well as neurocognitive impairment. This study aimed to determine whether specific bacterial species in the placenta of extremely preterm pregnancies are associated with neurological deficits later in life.

**Study Design:**

Using data from 807 children in the ELGAN study the risks of a low score on six neurological assessments in relation to 15 different microbes were quantified with odds ratios.

**Results:**

The presence of certain microbial species in the placenta was associated with lower scores on numerical and oral language assessments. *Lactobacillus* sp. was associated with decreased risk of a low oral language score and a composite measure of IQ and executive function.

**Conclusion:**

Placental microorganisms were associated with neurocognitive, but not social-communicative, outcomes at age 10. In contrast, the presence of the anti-inflammatory *Lactobacillus* sp. in the placenta was associated with a lower risk of impaired neurocognitive functions.

## Introduction

Children born preterm, before 37 weeks, are at a higher risk for neurocognitive impairment [1]. This impairment persists into school age and manifests as poor performance in school [2]. Studies have shown a higher incidence of intellectual deficit among children born preterm into adolescence [3, 4]. While most studies focus on intelligence quotient (IQ), children born preterm exhibit impairment in multiple domains of neurodevelopment including motor function [2], executive function [4], social cognition [5], language skills [6], and mathematical ability [7]. These cognitive deficits tend to co-occur in preterm children [2]. As prenatal care continues to improve the survival rates of preterm children, the number of these children living with neurological deficits and disabilities is increasing [8]. Thus, it is important to understand the etiology of neurocognitive impairment and the precursors of unfavorable cognitive outcomes in extremely preterm children.

The developmental origins of health and disease (DOHaD) hypothesis proposes that the prenatal environment can influence adult disease and later life outcomes [9], including neurocognitive and mental health [10, 11]. With relevance to DOHaD, the placenta is a critical regulator of the prenatal environment and is at the interface between the mother and developing fetus. The placenta transports nutrients from mother to fetus and produces hormones necessary to maintain pregnancy and support the fetus [12]. Once considered a sterile organ, the placenta has been found to harbor microorganisms [13]. The presence of certain bacterial species in the placenta has also been associated with pregnancy outcomes and fetal health [13, 14]. In addition, preterm birth has been associated with bacteria including *Ureplasma urealyticum*, *Mycoplasma hominis*, *Gardnerella vaginalis*, and *Peptostreptococcus* sp. in the placenta and intrauterine environment [14–16]. The presence of bacteria in the placenta is also associated with adverse neurological outcomes, especially in those born preterm [15, 17]. However, most of the studies that assessed neurocognitive outcomes used indicators of infection, such as chorioamnionitis, as opposed to directly testing the placenta for the presence of microbial species, a gap that will be addressed in this study.

For this study, we set out to examine neurocognitive and social-communicative function among school-age children in relation to placental bacteria. Previously within the ELGAN study it has been observed that children whose placenta harbored two or more microorganisms, compared to those with no or one microorganism, were at heightened risk of brain abnormalities detected by ultrasound and with forms of cerebral palsy two years later [18]. Specifically, the presence of *Ureaplasma urealyticum* was associated with increased risk of intraventricular hemorrhage and brain lesions in the white matter [19]. In the present study, we assess the neurological development of the ELGAN children at 10 years of age in relation to placental microbes. The goal of these analyses is to provide insights into whether microorganisms in the placenta are associated with long-term neurocognitive development and social-communicative behavior. Based on previous studies, we hypothesize that microorganisms in the placenta will be associated with differential performance and brain function of school-aged children.

## Materials and Methods

### ELGAN Study Recruitment and Participation

The recruitment process of the ELGAN study has previously been described [20]. Briefly, between 2002 and 2004, women who gave birth before 28 weeks gestational age at one of the 14 ELGAN research sites in the United States were asked to participate in the study. The Institutional Review Boards at each of the 14 study sites approved all procedures. Informed, written consent was provided within a few days before or after delivery. The mother’s consent covered both her and the child’s participation in the study.

A total of 1,249 mothers and 1,506 infants enrolled in the study of which 1,365 placentas were collected and analyzed for microorganisms. When children reached 2 years of adjusted age, 1102 participated in a neurodevelopmental assessment [20]. At the 10 year follow-up, 889 children were enrolled and evaluated. Of these children, 807 had their placenta parenchyma cultured and analyzed for microorganisms. These 807 participants made up the subcohort we included in this study.

### Clinical Data

A trained research nurse interviewed the mother within a few days of delivery to collect information about maternal sociodemographic factors. The research nurse reviewed maternal and neonatal medical records to collect additional clinical information [21].

### Placenta Sample Collection

Women participating in the ELGAN study were asked to provide their placentas for analysis. The placenta collection technique is as follows: delivered placentas were placed in a sterile exam basin and transported to a sampling room. The placenta parenchyma, which is located beneath the attached fetal membranes, was biopsied since the fetal membranes are likely to be exposed to sources of bacterial contamination during delivery. The sample was taken at the midpoint of the longest distance between the cord insertion and the edge of the placental disk. Using sterile technique, the amnion was pulled back to expose the chorion. Traction was applied to the chorion and a piece of the underlying trophoblast tissue was removed. The tissue was placed into a cryo-vial and immediately immersed into liquid nitrogen. Specimens were stored until processing at minus 80 °C [22].

### Bacterial Analysis of Placenta

Study placentas were biopsied following delivery and were assessed for microorganisms using methods common to clinical diagnostic laboratories as described previously [22]. Briefly, a sterile scalpel was used to remove a section of each placenta. The placental tissue was homogenized in a phosphate buffered saline solution (PBS) and serial dilutions of the homogenate were made in PBS. Aliquots of the original homogenate as well as the dilutions were plated onto selective and nonselective media, including: pre-reduced Brucella base agar, tryptic soy agar, chocolate agar, and A-7 agar. Following the incubation period various colony types were enumerated, isolated, and identified at the Brigham and Women’s Microbiology Laboratory using estimated criteria [23]. Since the constituents of the chorion parenchyma in the ELGANs study prevent the reliable detection of bacterial DNA by polymerase chain reaction techniques, this study assessed placental colonization patterns obtained only by culture techniques [24].

### Procedures for the Assessments at 10-years of Age

All families who participated in the 2 year follow up were contacted by mail and then by phone to invite them to participate in the 10 year follow up. Lost to follow up families were searched for on state vaccination registries, and other openly-available websites. Facebook was also used where approved by the local institution’s IRB.

Families willing to participate were scheduled for one visit during which all of the measures and assessments reported here were administered in three to four hours, including breaks (Table 1). While the child was tested, the parent or caregiver completed questionnaires regarding the child’s medical and neurological status and behavior.

**Table 1:** Description of neurocognitive assessment variables at 10 years of age

Measure Type	Assessment Variable	Description
Neurocognitive Measures	Latent Profile Analysis (LPA)	Includes 9 variables that assess IQ and executive function and categorize children into four neurocognitive groups: normal, low-normal, moderately impaired, and severely impaired
	Oral and Written Language Scales (OWLS) Oral Composite	Expressive and receptive language skills
	Wechsler Individual Achievement Test-III (WIAT-III) Numerical Operations	Academic function in mathematics
	WIAT-III Word Recognition	Academic function in word recognition
Social-communicative measures	Social Responsiveness Scale (SRS)	Identifies social impairment associated with autism spectrum disorder (ASD)
	Children’s Communication Checklist-2 (CCC-2) Pragmatic Language	Speech, vocabulary, sentence structure, and social language skills

### Neurocognitive Outcomes

The following four neurocognitive variables were selected to provide the most comprehensive information about cognitive and academic function. All assessments listed were conducted by the child during the 10 year follow up visit.

### Cognitive Function Derived from Latent Profile Analysis (LPA)

This outcome variable was derived from latent profile analysis of participants’ performances across nine measures of IQ and executive function, described in detail elsewhere [25]. IQ was assessed with the School-Age Differential Ability Scales-II (DAS-II) Verbal and Nonverbal Reasoning scales [26]. Executive function included two subtests from DAS-II, DAS Recall of Digits Backward and Recall of Sequential Order, which measured verbal working memory [26], and five subtests from the NEPSY-II (A Developmental NEuroPSYchological Assessment-II) [27]. The NEPSY-II Auditory Attention and Response Set measured auditory attention, set switching and inhibition, the NEPSY-II Inhibition and Inhibition Switching measured simple inhibition and inhibition in the context of set shifting, respectively, and the NEPSY-II Animal Sorting measured visual concept formation and set shifting. The LPA identified four subgroups of study participants with similar cognitive profiles: normal, low-normal, moderately impaired, and severely impaired.

### Oral and Written Language Scales (OWLS) Oral Composite

Expressive and receptive language skills were evaluated with the Oral and Written Language Scales (OWLS), which assess semantic, morphological, syntactic, and pragmatic production and comprehension of elaborated sentences [28]. The OWLS yields an oral composite score that includes both listening comprehension and oral expression. To correct for small differences in age at the time of assessment and to facilitate a comparison of our findings to those reported for term children we calculated Z-scores based on distributions of values reported for the historical normative samples that are described [28].

### Academic Function

Academic function was measured with the Wechsler Individual Achievement Test-III (WIAT-III) Word Recognition and Numerical Operations subtests [29]. For these tests, we again used Z-scores based on distributions of values reported for the historical normative samples [29].

### Social-Communicative Outcomes

The following two assessments were selected based on their association with autism spectrum disorder (ASD). Both variables are based on parent/caregiver-report assessments.

### Social Responsiveness Scale (SRS)

The SRS is a parent/caregiver based rating scale that identifies social impairment associated with ASD and quantifies its severity [30]. This 65-item instrument provides a total score reflecting severity of social deficits in the autism spectrum. Raw scores are converted to T-scores to account for gender and age differences [30].

### Children’s Communication Checklist-2 (CCC-2) Pragmatic Language

The Children’s Communication Checklist-2 (CCC-2) is a parent/caregiver based rating scale that was used to assess children’s pragmatic language skills [31]. The child’s pragmatic language ability is assessed with four CCC-2 subscales: Initiation, Scripted Language, Context, and Nonverbal Communication. For each child, we averaged the scaled scores for these four subtests to yield a CCC-2 pragmatic language composite score.

### Data Analyses

In order to determine whether placental microorganisms are associated with neurocognitive and social-communicative function at age 10, separate logistic regression models were performed for 15 bacteria species or groups isolated including: *Lactobacillus* sp., *Prevotella bivia*, *Gardnerella vaginalis*, anaerobic *Streptococcus*, *Peptostreptococcus* sp., *Escherichia coli*, alpha-hemolytic *Streptococcus*, *Ureaplasma urealyticum*, *Mycoplasma* sp., *Staphyloccocus* sp., *Propionibacterium* sp., *Actinomyces* sp., *Corynebacterium* sp., *Streptococcus* Group B, and *Streptococcus* Group D. Each model examined whether the presence of an individual bacterial species or bacterial type was associated with increased odds of scoring one or more standard deviations below the normative mean on five different assessments: OWLS Oral Language Composite, WIAT-III Word Recognition, WIAT-III Numerical Operations, SRS, and CCC-2 pragmatic language. In the case of LPA, the models examined whether the presence of an individual bacterial species or type was associated with increased odds of having moderate or severe cognitive impairment. Confounders included in the models were infant sex, gestational age, birth weight Z-score <−1, maternal education, antenatal steroid use, histologic inflammation of the chorion/decidua, and mother’s eligibility for government-provided medical-care insurance. Variables were classified as confounding if they displayed an association with both the exposure, placental microorganisms, and the outcome, neurocognitive function. To avoid over adjustment, variables that would lie along the causal pathway between placental microorganisms and neurocognitive function, including intraventricular hemorrhage, were not included in the models. These models yielded odds ratios (ORs) and 95% confidence intervals (CI) of each 10-year characteristic associated with the microbial organisms recovered from the placenta. Bacterial species were considered to be significantly associated with a neurological function if the OR 95% CI did not cross one, and *p*-value was <0.05. An organism was considered to be associated with an increased risk of preforming poorly on an assessment when the OR and 95% CI were above one. Conversely, a microorganism was considered to have a protective effect when the OR and 95% CI were below one. In addition, we conducted a sex-stratified analysis using the same models of the 15 bacterial species and the five assessments. The confounding factors in the sex-stratified models were the same except that infant sex which was excluded.

## Results

### Study Subject Demographics

The placentas of 807 of the 889 ELGAN subjects who participated in the 10-year follow-up assessment were analyzed for culturable microorganisms. These 807 individuals represented those for whom microbial placental data were available and make up our subcohort for this study. The subcohort is similar to the overall 10-year cohort as is demonstrated by similarities in the percentages across variables (Table 2). Within the subcohort there are slightly more males than females (51% versus 49%). Most of the children were born between 25 and 26 weeks (44%) while 278 (34%) were born during the 27^th^ week and 173 (21%) were born between 24 and 25 weeks. Sixty-five percent of mothers had private insurance; 35% had public insurance. Fourteen percent of mothers had completed 12 or fewer years of formal education, 48% completed more than 12 years but less than 16 years, and 35% completed 16 or more years. Ninety percent of mothers were treated with antenatal corticosteroids. Outcome data were missing from between 5% for OWLS Oral Language Composite scores and 2% for the WIAT-III Numerical Operation score and the LPA.

**Table 2:** Demographics

	Overall 10-year follow up (*n* = 889) *N* (%)	Subcohort of 10-year olds with placenta microbiology (*n* = 807) *N*(%)
Fetal sex		
Male	455 (51.2)	414 (51.3)
Female	434 (48.8)	393 (48.7)
Gestational age (weeks)		
24–25	187 (21.0)	173 (21.4)
25–26	400 (45.0)	356 (44.1)
27	302 (34.0)	278 (34.4)
Birth weight (Z-score)		
<−2	53 (6.0)	48 (5.9)
≥−2, <−1	120 (13.5)	107 (13.3)
≥−1	716 (80.5)	652 (80.8)
SES (insurance)		
Public	307 (34.5)	271 (33.6)
Private	568 (63.9)	524 (64.9)
NS	14 (1.6)	12 (1.5)
Maternal education (years)		
≤ 12 (high school)	126 (14.2)	112 (13.9)
Some college or associates degree	431 (48.5)	387 (48.0)
College or Higher	306 (34.4)	284 (35.2)
NS	26 (2.9)	24 (3.0)
Antenatal corticosteroids		
Yes	788 (88.6)	723 (89.6)
No	100 (11.2)	84 (10.4)
NS	1 (0.1)	0 (0)
Chorioamnionitis		
Yes	288 (32.4)	263 (32.6)
No	530 (59.6)	487 (60.3)
NS	71 (8.0)	57 (7.1)
LPA		
Yes	874 (98.3)	792 (98.1)
No	15 (1.7)	15 (1.9)
OWLS oral composite		
Yes	849 (95.5)	771 (95.5)
No	40 (4.5)	36 (4.5)
WIAT-III word recognition		
Yes	864 (97.2)	783 (97.0)
No	25 (2.8)	24 (3.0)
WIAT-III numerical operations		
Yes	874 (98.3)	792 (98.1)
No	15 (1.7)	15 (1.9)
SRS		
Yes	866 (97.4)	787 (97.5)
No	23 (2.6)	20 (2.5)
CCC-2 pragmatic language		
Yes	854 (96.1)	775 (96.0)
No	35 (3.9)	32 (4.0)

*NS* Not Specified

### Microorganisms cultured from study placentas

The most common bacteria cultured from the study placentas was *Staphylococcus* sp. which was detected in 94 (11.6%) placentas (Table 3). The least prevalent bacterial species detected was *G. vaginalis*, which was present in 28 (3.5%) placentas. The remaining bacterial species were found in between 36 (4.5%), in the case of *Streptococcus* Group D, and 64 (7.9%), in the case of *Corynebacterium* sp., placentas. These includes *U. urealyticum*, *Lactobacillus* sp., *E. coli*, and alpha-hemolytic *Streptococcus* which were detected in 43 (5.3%), 48 (5.9%), 49 (6.1%), and 53 (6.6%) placentas, respectively.

**Table 3:** Microorganisms detected in the placentas of 807 children born preterm and tested for neurocognitive outcomes at 10 years of age

Bacteria	*n* (%)
*Lactobacillus* sp.	48 (5.9)
*P. bivia*	41 (5.1)
*G. vaginalis*	28 (3.5)
Anaerobic *Streptococcus*	40 (5.0)
*Peptostreptococcus* sp.	49 (6.1)
*E. coli*	49 (6.1)
Alpha *Streptococcus*	53 (6.6)
*U. urealyticum*	43 (5.3)
*Mycoplasma* sp.	42 (5.2)
*Propionibacterium* sp.	55 (6.8)
*Actinomyces* sp.	47 (5.8)
*Corynebacterium* sp.	64 (7.9)
*Staphylococcus* sp.	94 (11.6)
*Streptococcus* Group B	38 (4.7)
*Streptococcus* Group D	36 (4.5)

### Association of neurocognitive and social-communicative function at age 10 in ELGAN placentas exposed to bacteria

Out of the four neurocognitive assessments analyzed, three (WIAT-III Numerical Operations, OWLS Oral Language Composite, and LPA of IQ and EF) were associated with statistically significant differential odds in relation to at least one microorganism. Neither of the social-communicative assessments displayed differential odds in relation to any of the microbial species that were cultured. In addition, the sex-stratified analysis showed no differential odds on any of the five assessments in relation to any of the microbial species (data not shown).

### WIAT-III numerical operation

**Fig. 1:**
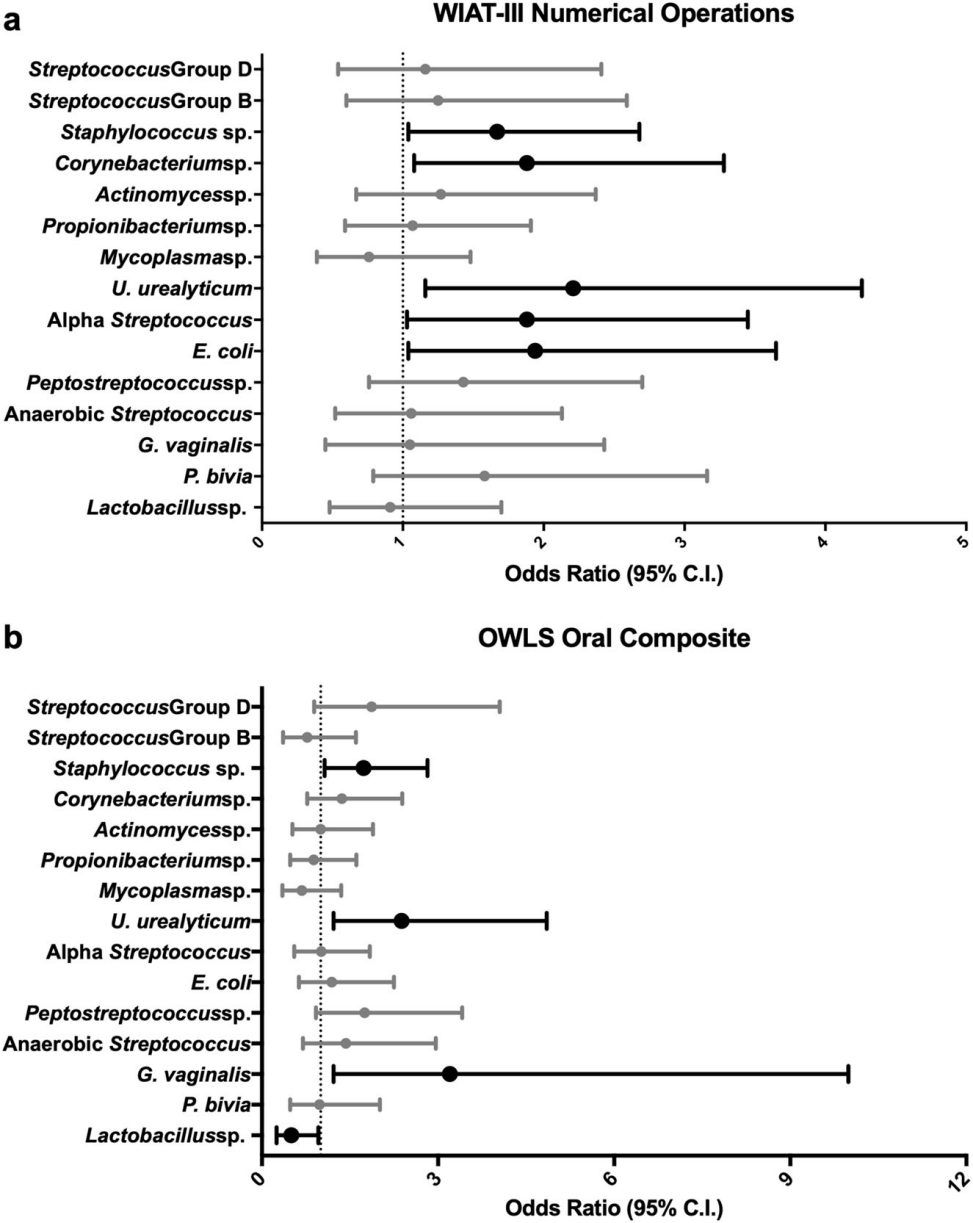
Odds ratios and 95% confidence intervals for WIAT-III Numerical Operations and OWLS Oral Composite in relation to placental microorganisms. The forest plots display ORs and 95% confidence intervals of a Z-score ≤ −1 on the (a) WIAT-III Numerical Operations subtest and (b) OWLS Oral Composite score at age 10 associated with the isolation of 16 bacterial species (y-axis). These odds ratios are adjusted for fetal sex, gestational age, birth weight Z-score, maternal education, public insurance, and antenatal corticosteroid use

For five of the 15 microorganisms detected, bacterial presence in the placenta was associated with increased odds of scoring one or more standard deviations below the normative mean on the WIAT-III Numerical Operations assessment (Fig. 1a). The strongest association was found with *U. urealyticum* (OR, 95% CI: 2.21, 1.16–4.26). Other bacteria associated with a low score on the WIAT-III Numerical Operations test were *E. coli* (OR, 95% CI: 1.94, 1.04–3.65), alpha-hemolytic *Streptococcus* (OR, 95% CI: 1.88, 1.03–3.45), *Corynebacterium* sp. (OR, 95% CI: 1.88, 1.08–2.68), and *Staphylococcus* sp. (OR, 95% CI: 1.67, 1.04–2.68).

### OWLS oral language composite

For three of the 15 microorganisms in the placenta, bacterial presence was associated with increased odds of scoring one or more standard deviations below the normative mean on the OWLS Oral Language Composite assessment (Fig. 1b). The strongest association was for *G. vaginalis* (OR, 95% CI: 3.20, 1.22–9.99). The other two bacteria associated with a low score on the OWLS were *U. urealyticum* (OR, 95% CI: 2.38, 1.22–4.85) and *Staphylococcus* sp. (OR, 95% CI: 1.73, 1.07–2.82). The presence of *Lactobacillus* sp. was associated with decreased odds of a low score on the OWLS (OR, 95% CI: 0.5, 0.25–0.96).

### Latent profile analysis

**Fig. 2:**
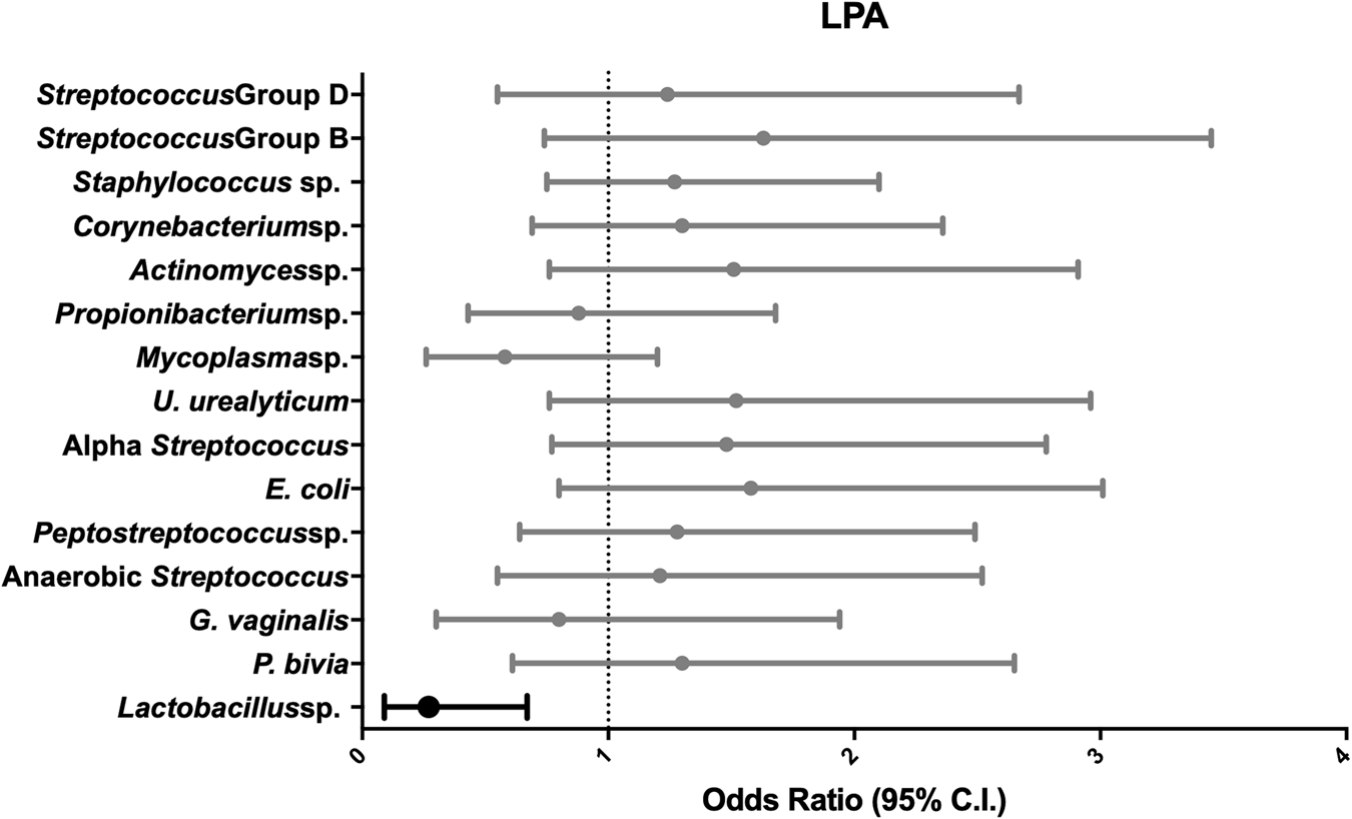
Odds ratios and 95% confidence intervals for LPA in relation to placental microorganisms. The forest plot displays ORs and 95% confidence intervals of a LPA subgrouping of severely or moderately impaired at age 10 associated with the isolation of 16 bacterial species (y-axis). These odds ratios are adjusted for fetal sex, gestational age, birth weight Z-score, maternal education, public insurance, and antenatal corticosteroid use

The presence of *Lactobacillus* sp. in the placenta was associated with decreased odds (OR, 95% CI: 0.27, 0.09–0.67) of having moderate or severe impairment as determined by LPA (Fig. 2). The other 14 microorganisms were not associated with LPA outcomes.

### *Lactobacillus* Interaction Analysis

In this analysis, *Lactobacillus* sp. was associated with lower odds of scoring low on the OWLS Oral Language Composite and lower odds of having moderate to severe cognitive impairment as characterized by LPA. Thus, we added an interaction term in the model between *Lactobacillus* sp. and the other 14 microorganisms to evaluate whether the effect of the interaction would significantly affect the ORs of our outcomes. However, we did not observe any significant effects in this analysis (data not shown).

## Discussion

In this study, several species of bacteria cultured from the placenta of children born extremely preterm were associated with neurocognitive impairments at 10 years of age. We demonstrate that bacteria associated with one or more adverse neurocognitive outcomes included *U. urealyticum*, alpha-hemolytic *Streptococcus*, *Corynebacterium* sp., *G. vaginalis* and *Staphylococcus* sp. In contrast, the presence of *Lactobacillus* sp. was associated with a lower risk of two adverse neurodevelopmental outcomes, general cognitive impairment and language deficit. Interestingly, bacterial presence in the placenta was not associated with social-communicative function.

In the ELGAN cohort, the recovery of alpha-hemolytic *Streptococcus*, *U. urealyticum*, or *G. vaginalis* from the placenta was associated with neonatal systemic inflammation in the first three postnatal days [32]. In contrast, recovery of *Lactobacillus* sp from the placenta was associated with a lower likelihood of neonatal systemic inflammation. These observations provide a plausible explanation for associations between placenta microorganisms and neurodevelopmental outcomes in the offspring, since neonatal systemic inflammation has been associated with neurodevelopmental impairments [33]. On the other hand Staphylococcal sp. (which in ELGAN were coagulase negative) were not associated with neonatal systemic inflammation, these organisms can cause sepsis, and presumably inflammation, in neonates [34].

Intrauterine and neonatal infection are strongly associated with negative birth outcomes, such as preterm birth [14], and also neurodevelopmental impairment [35]. In the present study, the presence of *U. urealyticum* in the placenta was found to be associated with deficits in language and mathematics. This same bacteria was previously associated with an inflammatory response in the chorioamnion, or chorioamnionitis [36], as well as in the amniotic fluid, cord blood, and fetal tissues [37, 38]. Providing further support for the present findings, the presence of *U. urealyticum* in the amniotic fluid has been found to be predictive of neuromotor delays at age 2 in a preterm birth cohort study [39]. Within the ELGAN cohort, *U. urealyticum* in the placenta has been associated with fetal and maternal inflammation, as well as white matter damage [19]. In addition to the findings with *U. urealyticum*, in the current study *E. coli* was associated with increased risk of performing poorly on WIAT-III Numerical Operations. This is interesting as in prior studies, exposure to *E. coli* increased the likelihood of white matter damage [40, 41]. White matter damage in newborns is predictive of a range of adverse neurodevelopmental outcomes including cerebral palsy, autism spectrum disorder, and psychiatric disorders [42–45].

In contrast to the other microorganisms, *Lactobacillus* sp. was associated with a decreased risk of oral language and general cognitive impairment, as measured by LPA. In certain contexts, *Lactobacillus* sp. has an anti-inflammatory effect; either by inhibiting NF-κB [46], a pro-inflammatory pathway, or by inducing the production of interleukin-10, an anti-inflammatory cytokine, in trophoblast cells [47]. In another ELGAN study, the presence of *Lactobacillus* sp. in the placenta was associated with a lower likelihood of neonatal systemic inflammation [32]. The genus *Lactobacillus* is composed of over 170 species and are taxonomically complex, making it hard to generalize about the genus [48]. In the ELGAN study *Lactobacillus* was not speciated further. Future research could speciate and determine whether particular species of *Lactobacillus* are causing this protective effect. Analyses to determine to what extent the presence of *Lactobacillus* sp. counteracts the effect of the other microorganisms in the placenta did not detect an interaction, perhaps because only a small number of placentas had *Lactobacillus* sp. present along with each of the other microbial types. For example, in only 1.4% of placentas was *Lactobacillus* sp. accompanied by *Staphylococcus* sp, the species found most often in our sample of placenta. No placentas harbored *Lactobacillus* sp. and either *U. urealyticum* or *Streptococcus* Group B.

While we have identified associations between microbes in the placenta and neurocognitive outcomes our study is not without limitations. There was a relatively low prevalence of each individual bacterium, limiting the power to detect associations. There is minimal chance that sample contamination could have contributed to the bacteria recovered, as has been reported for placenta samples analyzed by PCR and 16S sequencing [49, 50]. However, contamination by bacterial DNA would not affect the ability to culture bacteria. In addition, we specifically analyzed the placenta parenchyma to avoid contamination during delivery, and care was taken in the handling and processing of samples to avoid contamination of the placental tissue [24]. The bacteria recovered was not homogenous among samples, as would be expected if the bacteria originated from a contamination source. Furthermore, 387 (48%) of the placentas in this study harbored at least one type of microorganism, which is consistent with findings reported by others in both term and preterm placentas [13]. Strengths of this study are the large sample size, the broad range of assessments of neurocognitive and academic achievement, and the blinding of individuals who performed the neurodevelopmental assessments to information about placental microorganisms.

In summary, there were three major findings from this study. First, the presence of several different types of microorganisms in the placenta was associated with increased risk of learning limitations at age 10 among individuals born extremely preterm. Second, *Lactobacillus* sp. was associated with a lower risk of these learning limitations, information that could help inform intervention-based research and strategies. Finally, while placental microorganisms were associated with altered risk of neurocognitive outcomes they did not show an association with social-communicative function including autism. These results are relevant to the study of prenatal factors that influence neurocognitive function of children and provide further support to the DOHaD hypothesis linking the prenatal environment to health outcomes later in life.
